# Potential Application of Chimeric Antigen Receptor (CAR)-T Cell Therapy in Renal Cell Tumors

**DOI:** 10.3389/fonc.2020.565857

**Published:** 2020-09-23

**Authors:** Giuseppe Schepisi, Vincenza Conteduca, Chiara Casadei, Giorgia Gurioli, Lorena Rossi, Valentina Gallà, Maria Concetta Cursano, Nicole Brighi, Cristian Lolli, Cecilia Menna, Alberto Farolfi, Salvatore Luca Burgio, Amelia Altavilla, Giovanni Martinelli, Ugo De Giorgi

**Affiliations:** ^1^Department of Medical Oncology, Istituto Scientifico Romagnolo per lo Studio e la Cura dei Tumori, Istituto di Ricovero e Cura a Carattere Scientifico, Meldola, Italy; ^2^Biosciences Laboratory, Istituto Scientifico Romagnolo per lo Studio e la Cura dei Tumori, Istituto di Ricovero e Cura a Carattere Scientifico, Meldola, Italy; ^3^Unit of Biostatistics and Clinical Trials, Istituto Scientifico Romagnolo per lo Studio e la Cura dei Tumori, Istituto di Ricovero e Cura a Carattere Scientifico, Meldola, Italy; ^4^Department of Medical Oncology, Campus Bio-Medico University of Rome, Rome, Italy

**Keywords:** CAR (chimeric antigen receptor) T cells, RCC, immunotherapy, inflammation, toxicity

## Abstract

Currently, renal cell carcinoma is characterized by encouraging benefits from immunotherapy that have led to significant results in treatment outcome. The approval of nivolumab primarily as second-line monotherapy and, more recently, the approval of new combination therapies as first-line treatment have confirmed the importance of immunotherapy in this type of tumor. In this context, the chimeric antigen receptor (CAR)-T represents a further step forward in the field of immunotherapy. Initially tested on hematological malignancies, this new therapeutic approach is also becoming a topic of great interest for solid tumors. Although the treatment has several advantages over previous T-cell receptor-dependent immunotherapy, it is facing some obstacles in solid tumors such as a hostile tumor microenvironment and on-tumor/off-tumor toxicities. Several strategies are under investigation to overcome these problems, but the approval of CAR-T cell therapy is still some way off. In renal cancer, the significant advantages obtained from immune checkpoint inhibitors represent a good starting point, but the potential nephrological toxicity of CAR-T cell therapy represents an important risk. In this review, we provide the rationale and preliminary results of CAR-T cell therapy in renal cell malignancies.

## Introduction

Renal cell carcinoma (RCC) represents the 9th and 14th most common tumor worldwide in males and females, respectively. Incidence rates are higher in Europe and the United States than in Africa or South-Eastern Asia and are increasing in other countries especially in Latin America (1). Epidemiological studies have demonstrated that smoking, chronic kidney diseases, hypertension, and obesity are risk factors for RCC development (2). For decades, the only effective treatment against RCC was surgery because of its well-known chemoresistance. The subsequent approval of cytokines [interferon (IFN) and interleukin (IL)-2 (3)] and tyrosine kinase inhibitors (TKIs) led to an advantage in survival in patients with metastatic disease (4–7) [>26 months using vascular endothelial growth factor (VEGF) inhibitors (8)].

The approval of TKIs minimized the use of cytokines due to the differences in terms of survival and toxicity. Notwithstanding, the concept that IL-2 induces an immune response against tumor-mediated immune suppression (9) has, over the years, focused our attention on the possibility that RCC may be sensitive to immunological treatments. In recent years, the new Immunotherapeutic Era has led to the approval of several drugs for the treatment of urological tumors (including RCC) (3). Nivolumab (10), nivolumab + ipilimumab (11), pembrolizumab in combination with axitinib (12), and avelumab plus axitinib (13) have demonstrated the most important advantages in RCC in terms of survival and response with respect to TKIs (12–14). However, these advantages were obtained most frequently in two patient subgroups, defined as intermediate- and poor-risk cohorts according to the International Metastatic Renal Cell Carcinoma Database Consortium (IMDC). Research into the molecular reasons for this gap among patient cohorts is currently ongoing to drive clinical decisions.

More recently, the development of chimeric antigen receptors (CARs) has led to a new modality of immunotherapy. The astounding results in terms of responses demonstrated by this strategy against hematological neoplasms have turned the attention toward solid tumors, including RCC.

In this review, we will focus our attention on CAR-T cell therapy in patients with RCC.

### CARs: Structure and Function

**FIGURE 1:**
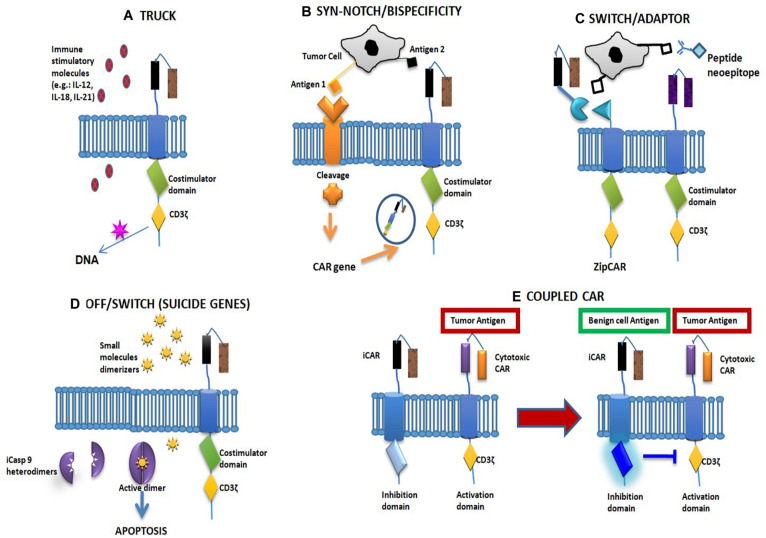
Fourth-generation chimeric antigen receptor (CAR) and main solutions to CAR toxicity. **(A)** The fourth-generation CAR structure. **(B,C)** CAR-related solutions. **(D)** Non-CAR-related solutions. **(E)** Coupled CAR. **(A)** The fourth CAR-T generation, also known as TRUCKs (T cells redirected for universal cytokine killing), is a CAR-T cell combined with immune stimulatory molecules such as cytokines [interleukin (IL)-12, IL-15, IL-18, IL-7R], multiantigen-targeting combinations [human epidermal growth factor receptor 2 (HER2), interleukin-13 receptor subunit alpha-2 (IL13Rα2), ephrin-A2 (EphA2)], knock-in genes (CXCR4, TRAC) and knock-out genes (PD1, DGK), or controlled and inducible systems (Syn/Notch). **(B)** “Synthetic Notch receptor”: the single-chain antibody fragment (scFV), connected with a Notch receptor fragment, is cleaved after antigen binding, allowing the intracellular domain to drive the expression of a second tumor antigen. This option improves tumor specificity of the CAR-T cells. Its double specificity controls off-tumor toxicities by depleting transferred cells or increasing specificity against tumor targets. **(C)** In “switchable CARs,” the use of adaptor molecules represents a tool to modulate CAR activation and longevity. The CAR molecule only binds a neoepitope, promoting T-cell activity after antibody–antigen binding. **(D)** The insertion of a truncated form of caspase-9 (iCasp9) with a binding domain specific for a “dimerizer” molecule could lead to a dimerization-related T-cell destruction in around 30 min. **(E)** Combination of a stimulatory CAR and an inhibitory CAR: the latter contains an intracellular domain with an immune checkpoint molecule (CTLA4 or PD1). In the event of contact with a normal tissue antigen, the inhibitory CAR irreversibly blocks T-cell activity.

Chimeric antigen receptor-T cells are T cells that are genetically engineered to express antigen-specific, non-major histocompatibility complex (MHC)-restricted receptors on their membranes. They are classified into four generations based on molecular complexity, all composed of different domains: (1) a single-chain antibody fragment (scFV) located in the extracellular part of the cell, representing the antigen-binding region; (2) a hinge domain linked to a (3) transmembrane region; and (4) an intracellular domain composed of the signal transduction part of the T-cell receptor (TCR), called the cluster of differentiation (CD)3ζ, linked with one (second and fourth CAR generations) or two (only third CAR generation) costimulatory domains. Moreover, the fourth CAR-T generation, also known as TRUCKs (T cells redirected for universal cytokine killing), are CAR-T cells combined with immune stimulatory molecules, such as cytokines [interleukin (IL)-12, IL-15, IL-18, IL-7R), multiantigen-targeting combinations [human epidermal growth factor receptor 2 (HER2), interleukin-13 receptor subunit alpha-2 (IL-13Rα2), and ephrin-A2 (EphA2)], knock-in genes, such as C-X-C chemokine receptor type 4 (CXCR4) and TCR α constant (TRAC), knock-out genes, e.g., programmed death-1 (PD1) and diacylglycerol kinase (DGK), or controlled and inducible systems, such as synthetic Notch receptor (Syn/Notch). Given that TRUCKs present both costimulatory element and proinflammatory factor, this characteristic increases T-cell efficacy with respect to previous CAR generations (Figure 1A). Those molecules have several advantages over the previous modalities of adoptive cell therapy, i.e., TCR and tumor-infiltrating lymphocytes (TIL) (15, 16). First and foremost, their immune activity is not MHC restricted because it is dependent on a surface–antigen interaction. This difference is crucial in treating tumors with low MHC expression, which, being TCR or TIL resistant, may be sensitive to CAR-T cell therapy (9, 17). Second, TCRs commonly have low antigen affinity, which can lead to off-target toxicities (18). Third, CAR-T cells not only have the antigen-binding activity of T cells (like monoclonal antibodies) but also their lytic property (19).

The first results of CAR-T cell therapy were obtained in hematological malignancies, where around 90% of complete responses were obtained in CD19-positive B-acute lymphoblastic leukemia. Conversely, only 26% of the patients with chronic lymphoblastic leukemia benefited from CD19 CAR-T therapy (20). This discrepancy seems to be related to the development of T-cell exhaustion induced by coinhibitory pathways. This exhaustion is, in turn, the cause of poor T-cell expansion and short-term persistence of T cells (21). In fact, by analyzing CAR-T cells from non-responders, an upregulation was found in pathways involved in exhaustion and apoptosis (22). In CAR-T cells, expression levels of T-cell coinhibitory receptors, e.g., PD-1, T-cell immunoglobulin and mucin domain-3 (Tim-3), and lymphocyte activation gene-3 (LAG-3) were found to be upregulated, probably to inhibit T-cell activity (23). For these reasons, some studies were conducted combining CAR-T cell therapy and immune checkpoint inhibitors (ICIs) in hematological malignancies, with interesting results (24).

### Challenges Facing the Use of CAR-T Cells in Treating Solid Tumors

Chimeric antigen receptor-T cell therapies were initially developed for the treatment of hematologic neoplasms but have not shown the same efficacy in solid tumors. In the latter, better results have been obtained from the use of TIL-dependent immunotherapeutic agents. This discrepancy is due to several conditions: (a) immune-mediated tumor antigen selection, which can improve the proliferation of tumor cells whose membrane does not express that specific target (25); (b) poor intra- and peritumoral trafficking (26); (c) limited CAR-T persistence in the host (27, 28). Little is also known about potential surrogate markers of T-cell persistence. One study on RCC patients demonstrated a correlation between CAR-T persistence and IFN-γ and IL-6, whereas the same correlation was not shown in relation to CAR-T toxicities (29); (d) CAR-T destruction mediated by the hostile tumor microenvironment (30); and (e) absence of a cancer-specific antigen in several tumor types that is suitable for inclusion in the CAR structure (31). This relative aspecificity may increase the risk of immune-related toxicity, as recently reported in literature (31–34).

### CAR-T-Related Toxicities

Several CAR-T-related toxicities have been reported. In particular, this therapy may induce severe toxicities, potentially affecting kidney function, including cytokine release syndrome (CRS), acute kidney injury (AKI), and tumor lysis syndrome (TLS). These toxicities are of primary importance for all cancer patients, especially for RCC patients, who have often undergone nephrectomy.

Several trials on hematological malignancies reported that CRS occurred in 40–50% of the patients. Its characteristic signs were that of hyperpyrexia for about a week followed by organ dysfunction (with cardiac failure in around 25% of the cases); high levels of C-reactive protein (>20 mg/dl) and ferritin; and hypotension, hypoxia, and neurologic symptoms, such as obtundation and seizures (35). This problem is related to higher levels of IL-6, which determines vasodilation, hypotension, hypoperfusion and, consequently, AKI. The fundamental role of IL-6 in the development of this syndrome has also been confirmed by the fact that the use of the anti-IL-6 receptor antibody tocilizumab restored organ function in several cases (36) in whom an alteration in electrolyte levels was frequently observed. However, it is still not clear whether this was directly due to CAR-T cell therapy or CLS.

Tumor lysis syndrome, another syndrome potentially related to the use of CAR-T cell therapy, is characterized by an elevation of lactate dehydrogenase and uric acid levels around 3 weeks after CAR-T infusion (37).

Chimeric antigen receptor-T cell therapy can also cause other non-renal adverse events, such as neurological and “on-target/off-tumor” toxicity. The former, as previously stated, is characterized by seizures, confusion, myoclonus, delirium, and expressive aphasia. These symptoms have been reported in patients specifically treated with a CD19-specific CAR-T, and it is still not known whether they could be caused by other antigen-specific treatments. Neurological toxicity is probably related to CRS but may also be related to a central nervous system-directed toxicity.

“On-target/off-tumor” toxicity is linked to antigen engagement in non-cancer tissues. First demonstrated in hematological malignancies, it has also been reported in solid neoplasms, such as gastrointestinal and lung cancer. More recently, the use of a carboxyanhydrase-IX (CAIX)-specific CAR-T cell for RCC caused the development of cholestasis because of CAIX expression in biliary duct epithelium (38, 39). This toxicity seems to be correlated with dosage, as shown in cases of HER-2-specific CAR-T infusion: high doses have led to patient death (33), but lower doses have proven safe (40).

Anaphylaxis and graft-versus-host disease are toxicities described in CAR-T cell trials. The former is often due to the use of murine domains in developing CAR molecules (36, 41). For the latter, two types of strategies are being pursued to counteract the potential alloreactivity linked to the infusion of non-host CAR-T cells, CAR-transduced viral-specific cells and endogenous TCR silencing (42–44).

### Potential Solutions to Reduce CAR Toxicity

As mentioned above, CAR-related toxicity represents an important limit to the development of this strategy in solid tumors, and several studies are ongoing to evaluate potential solutions to the problem (45). These can be divided into three groups: (1) CAR related, (2) non-CAR related, and (3) coupled CARs.

The first group comprises studies exploring the possibility of modifying the CAR structure to avoid toxicity. For example, it has been demonstrated that designing a CAR with a reduced antigen affinity in its scFV can spare normal tissues from immune-mediated consequences (46–48). The same effect has been achieved by modifying the extramembrane spacer length between the scFV and the cellular membrane (18, 49). Another option consists in the development of a “synthetic Notch receptor”: the scFV, connected to a Notch receptor fragment, is cleaved after antigen binding, allowing the intracellular domain to drive the expression of a second tumor antigen. This option improves the tumor specificity of CAR-T cells (49) (Figure 1B). Other options included in this group are the development of “split CARs” in which the CAR domains are only linked in the presence of a small molecule with dimerizing activity and the development of “switchable CARs” in which the CAR molecule only binds a neoepitope that can activate T-cell activity after antibody–antigen binding (50, 51) (Figure 1C).

The second group is composed of solutions not involving the CAR structure but which act at different levels. For example, some studies have inserted inducible suicide gene cassettes to induce apoptosis in the T cell in cases of immune-related toxicity. Two strategies have been tested: (a) the insertion of a truncated form of caspase IX (iCasp9) with a binding domain specific for a “dimerizer” molecule, which can lead to a dimerization-related T-cell destruction in around 30 min (52) (Figure 1D) and (b) the inclusion of the gene for herpes simplex virus tyrosine kinase. In this case, the administration of ganciclovir can induce T-cell apoptosis (53). Using a similar procedure, an “elimination gene” can also be inserted, leading to antibody-mediated T-cell destruction (in the event of toxicity) (54).

The third group is composed of studies evaluating the possibility of using two CAR molecules with different functions: (a) a combination of a CAR with an intracellular TCR portion, while the stimulatory portions are located on the second CAR. The T-cell immune activity can only start when both CARs bind their antigen. Results, to date, have been somewhat contradictory, and in one study this, procedure was also shown to destroy healthy cells expressing only one antigen (52, 55); (b) a combination of a stimulatory CAR and an inhibitory CAR: the latter contains an intracellular domain for an immune checkpoint molecule (CTLA4 or PD1). In the event of contact with a normal tissue antigen, the inhibitory CAR blocks T-cell activity. This block is reversible, and so T cells can be reactivated by another tumor antigen (56) (Figure 1E).

### Inflammation, CAR-T Treatment, and Its Rationale in RCC

The role of immunotherapy has always been considered in RCC, and this approach has obtained significant results from the IL-2 era to the latest ICIs. The rationale for its efficacy lies in the involvement of the immune system in RCC, and several studies have investigated this correlation. Trials investigating the role of ICIs in RCC patients demonstrated a >1% PD-L1 expression ranging from 24% in the CheckMate-214 study (57) to 63.2% in the JAVELIN Renal 101 study (13). In both trials, the overall response rate (ORR) and survival (OS) were sharply in favor of immunotherapy, especially in the presence of PD-L1 expression. The JAVELIN Renal 101 study demonstrated a 55.2% ORR from the combination of avelumab plus axitinib compared to 25.5% from sunitinib (the data reported were only from PD-L1-positive cases). Conversely, the combination of nivolumab + ipilimumab in the CheckMate-214 study showed a 42% ORR compared to 26% in the sunitinib arm. In the same study, the immunotherapy combination obtained a median OS gain of 20 months compared to the sunitinib arm (47 versus 26.6 months, respectively) (58).

Both studies highlighted a difference between the IMDC prognostic groups, showing that the intermediate- and high-risk groups tended to benefit more from immunotherapy, contrary to what happens in good prognosis cases, which appear to do better with oral TKIs (58). Recently, McDermott et al. evaluated the impact of immunotherapy according to the IMDC risk criteria, selecting a heatmap of genes previously established as angiogenesis-related and immune biology-related genes. They identified subgroups with different biological features, the differences based on the expression (high/low) of angiogenesis (Angio), immune system (T effector), and myeloid inflammation-associated genes. The subgroup expressing a high T-effector gene signature responded best to immunotherapy, whereas the cases with a high Angio signature benefited more from TKIs. Patients with a high myeloid gene signature treated with immunotherapy showed a poorer survival than those with a low expression of the gene (59).

In parallel to this type of study, the correlation between the immune system and RCC was also assessed from a prognostic/predictive point of view. In the cytokine era, absolute neutrophil count was considered as a prognostic factor in a prognostic model for RCC validated by the Groupe Francais d’Immunotherapie (60). Subsequently, several studies validated the role of other inflammation parameters such as neutrophil/lymphocyte ratio (NLR) (61), systemic inflammation index (SII) (62), and CRP (63) as independent prognostic factors in RCC. With the discovery of inhibitory checkpoints, greater attention was paid to more specific prognostic and predictive markers. The PD1/PD-L1 axis is also the most widely studied parameter in RCC. PD-L1 expression is known to be significantly related to poor response to antiangiogenic treatments and has shown an independent association with shorter survival in stage IV RCC pretreated with VEGF–TKIs (64, 65). Overall, PD-1, PD-L1, and PD-L2 expression is associated with poor outcome in TKI-pretreated RCC patients (66). As mentioned above, the most recent studies on ICI combinations as first-line treatment reported better patient outcomes than those on sunitinib in intermediate and poor prognostic groups, suggesting a correlation between prognosis and “immuno-susceptibility” in RCC patients (14).

Interest has recently been aroused in the role of inflammasome complexes in solid tumors. These multimolecular complexes are known for their ability to control the activation of caspase-1, a proteolytic enzyme involved in the maturation of proinflammatory cytokines (IL-1β and IL-18), and in inducing inflammatory-like apoptosis (pyroptosis) against pathogens and endogenous danger signals. In brief, inflammasome may play a role in neoplastic development via the regulation of tumor inflammation (67). Some studies on RCC have shown that enhancing inflammasome activation blocks tumor proliferation, promoting pyroptosis. Wang et al. demonstrated that the nuclear receptor liver X receptor alpha (LXRα) is upregulated and associated with a poor prognosis in RCC patients (68). In fact, LXRα downregulates the NLRP3 inflammasome, leading to metastatization. Tan et al. showed that tumor proliferation and epithelial mesenchymal transition (EMT) in RCC patients are inhibited by BRD4 inhibition (69). The authors demonstrated that this molecule, an epigenetic reader, exerts an antitumor effect by activating pyroptosis. Chai et al. reported that absence in melanoma-2 (AIM-2), a tumor suppressor, influences inflammasome activity in RCC (70).

Based on what has been reported so far, especially for the role that the modulation of the immune system has always shown in the treatment of renal neoplasms, it is clear that RCC represents one of the most interesting test beds for the development of CAR-T technology in solid tumors.

### CAR-T Cell Therapy in RCC Patients: Pros and Cons

At this point, a list of pros and cons can be compiled on the development and subsequent use of this new therapeutic approach in patients with RCC. It is undoubtedly an innovative and interesting therapy because of its high response rates obtained in hematological diseases and would also appear to be a promising strategy in RCC patients. It is a non-MHC-restricted approach and so has several advantages over TCR, as previously mentioned. Furthermore, unlike MHC-restricted immunotherapy, CAR-T cell therapy is susceptible to the modulation of T-cell function to improve efficacy and reduce toxicity. This last aspect is both an advantage (the ability to self-modulate antitumor activity is certainly an improvement compared to the past) and a disadvantage because, as previously mentioned, this type of treatment can cause particularly severe toxicities that were not induced by previous therapeutic approaches. In particular, the risk of renal toxicity, such as AKI, must be accurately evaluated in RCC patients who frequently undergo nephrectomy. We must also not forget the high cost of the drug, as well as the time required (a few weeks) for its preparation. It may not always be possible to wait so long before starting a treatment. In addition, CART-T cell therapy requires apheresis and adequate lymphocyte count and function, which may exclude some patients.

For these reasons, if an efficacy of CAR-T cell therapy similar to that observed in hematological malignancies is proven in RCC, the pros and cons of its use will need to be carefully evaluated in each individual patient.

### CAR-T and Radiotherapy in RCC Patients

The association of immunotherapy with radiation therapy has been under investigation for some time, some studies hypothesizing its potential usefulness for the treatment of different cancers, including RCC (71–74).

The recent development of CAR-T cell therapy in solid tumors has led to the hypothesis of its combination with radiation therapy. In fact, the latter would appear to play a role in stimulating cancer antigenicity, promoting CAR-T cell chemotaxis and making the tumor microenvironment more sensitive to immune activation (75). In particular, it has been shown that γ-irradiation can enhance CAR-T efficacy by increasing tumor antigen expression on cancer cell surface and by stimulating IFN-γ secretion. Secreted by cancer cells, IFN-γ is known to promote immune infiltration into the tumor microenvironment, and radiation therapy influences tumor vasculature, facilitating the diffusion of lymphocytes within the tumor (75). However, there are still very few data on the combination of radiotherapy and CAR-T cell therapy in RCC. Given the solid rationale for this combination, further research is warranted.

### CAR-T Cell Therapy: Ongoing Trials in RCC

In recent years, several articles have been published on the role of CAR-T cell therapy in solid tumors. Some studies are ongoing in the area of RCC (Table 1): a dose escalation and dose expansion trial is being carried out to assess the efficacy of autologous CAR-T cells CCT 301-38 or CCT 301-59 in recurrent/refractory stage IV RCC. The authors are simultaneously evaluating the effectiveness of two CARs directed against two different molecular targets. CCT301-59 is a CAR-targeting tyrosine kinase-like orphan receptor 2 (ROR2), an atypical receptor of the tyrosine kinase family involved in several human diseases. In RCC patients, ROR2 expression is correlated with other genes associated with mytosis and migration, including PCNA, CDK1, TWIST, and MMP-2 (76). CCT301-38 is another CAR directed against AXL, a cell surface tyrosine kinase receptor, which is part of the TAM kinase family. AXL, the high-affinity ligand growth arrest-specific protein 6 (GAS6), is involved in multiple tumor processes, including proliferation, angiogenesis, invasion, metastatization, immune regulation, stem cell maintenance, EMT, and drug resistance. Aberrant Gas6/AXL expression has been described in several tumor types, including RCC (77).

**TABLE 1:** Clinical trials of chimeric antigen receptor (CAR)-T cell therapy in renal cell carcinoma (RCC).

ClinicalTrials.gov Identifier	Study Title	Locations
NCT03393936	Safety and Efficacy of CCT301 CAR-T in Adult Subjects With Recurrent or Refractory Stage IV Renal Cell Carcinoma	Shanghai Public Health Clinical Center, Shanghai, China
NCT01218867	CAR T Cell Receptor Immunotherapy Targeting VEGFR2 for Patients With Metastatic Cancer	National Institutes of Health Clinical Center, Bethesda, MD, United States
NCT02830724	Administering Peripheral Blood Lymphocytes Transduced With a CD70-Binding Chimeric Antigen Receptor to People With CD70 Expressing Cancers	National Institutes of Health Clinical Center Bethesda, MD, United States
NCT03638206	Autologous CAR-T/TCR-T Cell Immunotherapy for Malignancies	The First Affiliated Hospital of Zhengzhou University, Zhengzhou, Henan, China
NCT04438083	Phase 1 study evaluating the safety and efficacy of CTX130 in subjects with relapsed or refractory renal cell carcinoma.	CRISPR Therapeutics AG, Melbourne, VIC, Australia

In the ongoing trial, patients with a ROR2-positive biopsy will receive CCT301-59, while those with an AXL tyrosine kinase receptor-positive but ROR2-negative biopsy will receive CCT301-38. A blood sample will be taken from patients to isolate peripheral blood mononuclear cells (PBMCs) for the production of CCT301-48 or CCT301-59. During the procedure, patients will undergo a cyclophosphamide plus fludarabine conditioning regimen to deplete lymphocytes, after which one intravenous cycle of CCT301-48 or CCT301-59 will be administered. A 3 + 3 dose escalation model will investigate the safety and efficacy of these molecules. Three different CAR T dosages will be evaluated: 1 × 10^5^/kg, 1 × 10^6^/kg, and 1 × 10^7^/kg CAR+ T cells. This study is currently active but not recruiting patients. The primary completion date of the study is scheduled for the first months of 2021 (NCT03393936).

Another study is evaluating the safety and effectiveness of the anti-VEGFR2 gene-modified CD8 cells in mRCC patients (NCT01218867). Patients undergo lymphocyte-depleting chemotherapy (cyclophosphamide and fludarabine) followed by CAR gene-transduced CD8+ PBMC in combination with aldesleukin. Participants are divided into two cohorts according to histology: cohort 1 includes metastatic melanoma and RCC patients and cohort 2, patients with other metastatic tumor types. Preliminary results have failed to show any objective responses.

The same lymphodepleting preparative regimen is also being used in another study currently ongoing to test peripheral blood lymphocytes transduced with anti-hCD70 CAR in combination with aldesleukin. CD70, normally expressed in B, T, and NK cells, is a transmembrane receptor with a costimulatory role in immune cell activation. CD70 is upregulated in several tumors where it stimulates immune escape by promoting cytotoxic effects on B and T lymphocytes. It is highly expressed in different RCC histologies (clear cell, sarcomatoid, and some papillary tumors), and this condition is correlated with decreased survival, thus representing a potential target for therapies against RCC.

Two separate cohorts will be included, one with patients with CD70-expressing RCC and another with patients with CD70-expressing non-RCC solid tumors (NCT02830724).

More recently, a phase I study has begun enrollment of patients with advanced, relapsed, or refractory clear-cell RCC to test a new allogeneic therapy (CTX130). CTX130 consists of an allogeneic CRISPR/Cas9 gene-edited CAR-T cell therapy targeting CD70, which is currently under development for the treatment of both solid tumors and hematologic malignancies. The theoretical advantages of this type of CAR (allogeneic) are: (1) immediate availability; (2) increased potency (because it is derived from healthy donors); (3) greater consistency (many doses from healthy donors); (4) no need for patient apheresis; and (5) flexibility to titrate dose or re-dose. The final data on the primary outcome measure is expected in February 2027 (NCT04438083).

Overall, despite the interesting ongoing research, conclusive results have yet to be obtained.

## Conclusion

Unlike other genitourinary tumor histotypes, RCC is a neoplasm for which numerous drugs have been approved. After decades in which no effective therapies were available because of its known chemoresistance, the advent of biological therapies and then immunotherapy a little over a decade ago has brought about a marked improvement in terms of survival. In the wake of these data, the unexpectedly promising results obtained from CAR-T therapy in hematological tumors has prompted research into the possibility of also using this treatment for RCC.

To date, however, there are still few results from few studies. Furthermore, the peculiarities of solid tumors pose different challenges with respect to hematological malignancies. The presence of stroma and various inhibitory factors within the tissue, the problems related to T-cell trafficking, and the non-high antigenic selectivity represent just a few of the obstacles to the successful outcome of the treatment. In addition, the risk of side effects, including the aforementioned “on-target/off-tumor” toxicity justifies the delay in the use of CAR-T cell therapy in solid tumors, including RCC. Further research is thus needed to resolve these problems before being able to claim an efficacy comparable to that achieved in hematological tumors.

## Author Contributions

GS and UD conceived and designed the article. GS created the table and figures and drafted the manuscript. CC was responsible for compiling the “References” section. VC, GG, LR, VG, MC, NB, CL, CM, AF, SB, AA, and GM analyzed the data. All authors read and approved the final version of the manuscript for submission.

## Conflict of Interest

The authors declare that the research was conducted in the absence of any commercial or financial relationships that could be construed as a potential conflict of interest.
